# Physician gender as a source of implicit bias affecting clinical decision-making processes: a scoping review

**DOI:** 10.1186/s12909-021-02601-2

**Published:** 2021-03-19

**Authors:** Tiffany Champagne-Langabeer, Andrew L. Hedges

**Affiliations:** School of Biomedical Informatics, The University of Texas Health Science Center, 7000 Fannin Street, Suite 600, Houston, TX USA

**Keywords:** Implicit bias, Gender medicine, Provider characteristics, Medical education

## Abstract

**Background:**

The demographic profile of practicing physicians is changing as more female medical students are graduating and practicing in the field. While the education received may not differ by gender, studies have shown that physician practice outcomes vary by provider gender. Various factors could contribute to these differences, including culture and explicit biases which may lead to implicit bias. This study aims to identify the available evidence of gender-based implicit bias throughout the delivery process of medicine.

**Methods:**

This scoping review evaluated published literature within the PubMed, Ovid MEDLINE, PsychINFO, Web of Science, and BioMed Central databases pertaining to physician’s gender as a factor in the delivery of medicine. Arksey and O’Malley’s six-stage methodology was used as a framework and reported using the updated Preferred Reporting Items for Systematic reviews and Meta-Analyses extension for Scoping Reviews (PRISMA-ScR). Searches occurred between May 2020 and June 2020, and the timeframe was not limited. Included articles had gender as a factor in the delivery of medicine and implicit bias. Articles were excluded if they did not include the gender of the physician. After screening by reviewers and a medical librarian, study characteristics were charted and analyzed.

**Results:**

The initial search resulted in 2420 records. After screening, 162 of the records were selected based on title and keyword relevance. After additional screening, 15 records were ultimately included in the review based on full-text evaluation. Records were organized into sub-topics post hoc focused on clinical qualities, diagnostics, treatment, and outcomes.

**Conclusion:**

This scoping review found that gender-based implicit bias may be inadvertently acquired from culture and education. Although implicit bias is highly researched, much of the current literature focuses on the gender of the patient. This study found important gaps in the available literature regarding race and gender of the physician. Further studies could explore outcome differences between recent graduates and career physicians, for both female and male physicians.

**Supplementary Information:**

The online version contains supplementary material available at 10.1186/s12909-021-02601-2.

## Introduction

Undergraduate medical education has experienced a plethora of new challenges which pose a significant social impact on the practice of medicine. Society places an unspoken moral contract on the profession of physicians, and the concept of medicine as both *art and science* further emphasizes the subjective nuances of care delivery [1]. Undergraduate medical education intertwines this sentiment defining medicine as “… a profession that incorporates science and the scientific method with the art of being a physician” [2]. There is a qualitative component of expertise which is indicated by the social skills involved in physician-patient interactions, and an intuitive sense involved in diagnostic decision-making. Within the modern healthcare environment, good medical practitioners understand how crucial and inseparable both aspects can be in patient outcomes [3]. Initiatives to demonstrate patient-centeredness require that physicians exercise their artistic capabilities while maintaining evidence-based practice guidelines [4]. This is partially in hopes of lowering health disparities affecting disenfranchised patients; however, evidence shows that many disparities persist today [5, 6]. Several factors contribute to the presence and persistence of these disparities; however, it remains a challenge to promote awareness of bias while not assigning blame [7]. While it is the rigor of science and research that develops clinical practice guidelines, it is the art and judgment of an individual physician which impacts the patient.

Within the context of healthcare, the concept of *prejudice*, or perceived feelings regarding a patient based upon certain characteristics such as race, gender, ethnicity, sexual orientation, or other variables, is considered negative and is discouraged within the medical profession [8, 9]. However, implicit bias–defined as preferential associations that exist subconsciously—is subtle and harder to detect [10]. In psychological terms, implicit bias can be viewed as a latent or subconscious construct. In this way, a physician with an (unrealized) implicit bias against patients who use heroin may create preconceived judgments about the patient’s ability to adhere to medication. This may or may not change their behavior towards the patient. Thus, implicit bias may be described in terms of the resultant behavior: the decision made by the physician [11]. There are assessments for measuring implicit bias such as the Implicit Assessment Test (IAT); however, this assessment intends to measure the deeply held beliefs which do not necessarily translate into action [12, 13]. The IAT in vignette-based studies has shown mixed results, with implicit bias correlating to action or having no relationship to outcomes [14]. The definition by De Houwer and colleagues of implicit bias as a “behavioral phenomenon” may prove more useful, as measuring a subconscious and often unrealized construct in the absence of action proves challenging and often unreliable [11].

Implicit bias may be observed through the actions of physicians as well as patients. Prior studies have shown some medical professionals have a preference for others who resemble their own ethnicity and skin tone [15]. Similarly, African American patients who feel disenfranchised by the healthcare system, prefer a physician of their own race [16]. Akin to implicit bias, the fear of stigma may cause lesbian, gay, bisexual, and transgendered (LGBT) patients to withhold certain information from caregivers due to mistrust of medical providers or fear of discrimination [17, 18].

Medical schools graduate more female physicians than at any time before in their histories, which brings forth the topic of gendered medicine [19]. Many institutions and medical centers hire more female physicians, rejecting inaccurate, institutionalized explicit biases [20]. However, women persist in small numbers in positions of leadership, especially at the executive or dean level with only 16% representation [21]. There are negative implications to an unbalanced leadership hierarchy. Organizational structures tend to perpetuate themselves; therefore, without conscious interventions in place, men are promoted more often than women [22]. The secondary effect on the medical student population is one which projects the subliminal message of who is accepted and most likely to succeed [23]. A dearth of females in positions of authority or influence can reduce the potential for scientific discovery, as females are more likely to develop new programs related to women’s health [24, 25]. Prior research has addressed the impact of increased diversity and specific metrics were reviewed as more favorable when female physicians were caring for patients within management [26] and overall wellbeing [27]. However, due to the subjective nature of clinical decision-making, no consensus on a primary determinant exists for the differences seen in real-world medical practice [28].

The impact of gender on medical decision-making has traditionally focused on the physicians’ perceptions of their patients. A review of research in cardiovascular health highlights differences in the clinical presentation, subsequent diagnosis, and reveals the existent gender-gap in patient outcomes in sophisticated modern health systems [29, 30]. Differences in the management of cardiovascular health also show gender-based biases when making decisions for treatment, but the results are mixed. In one study, both male and female physicians performed fewer cardiac catherizations on female patients; however, the same study showed that female physicians performed fewer procedures in general when compared with male physicians [31]. In a separate study, male patients were seen as strong and greater value was placed on stress tests and angiography, while female patients were seen as risk-averse by both male and female cardiologists [32]. The reasons for treatment variations not explained by gender are valid differences in clinical presentation between men and women including women have a higher incidence of ischemic stroke; whereas, men are more prone to myocardial infarctions [33]. Aside from clinical presentation, some studies have attributed downstream differences in physician behavior to the training received in medical school, residency, internships, and other phases in medical education [34], while others assert the workplace as a contributing factor [35]. A review of the research shows the impact of patient gender on clinical decision-making and the implicit biases associated are multifactorial.

In the medical field, the downstream effects of negative implicit bias can contribute to increased medical costs or death [27]. To date, much of the research related to implicit bias occurring within healthcare has focused on the gender of the patient as opposed to the gender of the physician. And while many within the medical field recognize gender-based differences between the practices of male and female physicians, no comprehensive review exists that explores the breadth of these provider differences and the origin of gender-based implicit bias. This scoping review attempts to answer two questions: 1) what implicit biases exist between male and female physicians; and 2) how do the coinciding implicit biases manifest in clinical behavior, performance, and outcomes. This scoping review aims to address the lack in available knowledge by coalescing existing publications and perspectives, in hope of describing gender-based clinical decision-making within the physicians’ daily practice.

## Methods

### Record selection

**Fig. 1:**
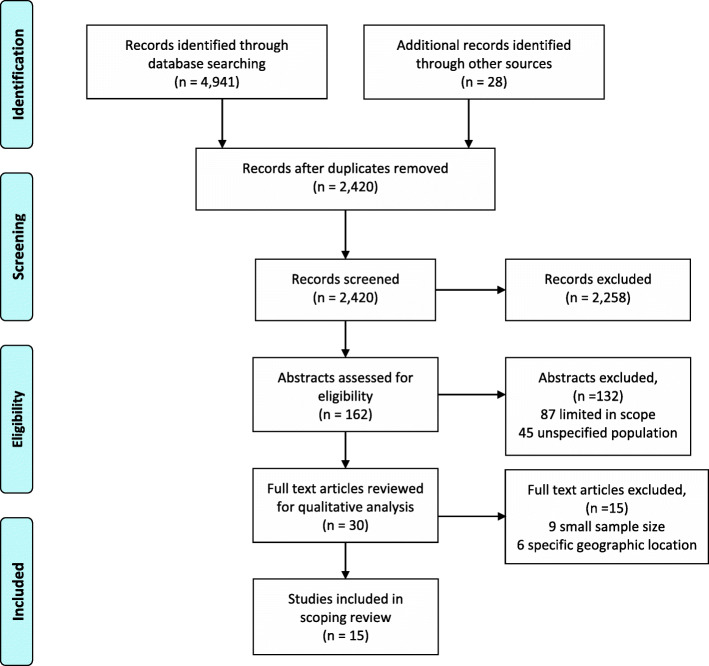
Article Search and Review Process

In contrast to systematic reviews, scoping reviews address gaps in knowledge while developing additional research questions within a particular topic of interest [36]. This research followed Arksey and O’Malley’s framework [37] and was reported using the updated Preferred Reporting Items for Systematic reviews and Meta-Analyses extension for Scoping Reviews (PRISMA-ScR) checklist [38]. The methodology was established in a stepwise manner. First, the research questions were identified. Next, a medical librarian assisted in identifying relevant search terms, exploring research databases, and refining the criteria for record inclusion. Third, a review of abstracts of relevant, selected records occurred. Articles included were available in the English language. (Fig. 1). Next, the selected full-text articles were reviewed, charted, and the results synthesized. Lastly, the studies were organically grouped based on relevance to various subtopics in order to illuminate insights from the literature.

The initial strategy for finding relevant published records included use of the PubMed, Ovid MEDLINE, and PsychINFO databases. Secondary searches followed within BioMed Central and the Web of Science databases. A large range of search terms were needed to define relevance criteria, both during the search and while evaluating the accompanying results. Select keywords and phrases included: ‘provider sex’, ‘physician sex’, ‘practice patterns’, ‘clinical decision making’, ‘gender concordance’, ‘sex-based differences’, and ‘implicit bias’. A complete list of terms in included in Appendix A. These searches occurred between May 2020 and June 2020, and the timeframe of when the articles were published was not limited.

Both authors reviewed abstracts from the keyword and medical subject heading (MeSH) search then met to determine articles for full text review. Both authors reviewed the subsequent full text selection, and disagreements were handled by a third reviewer (the medical librarian). After completing the selection process for inclusion in the next stage, the level of relevance for the resulting records occurred by assessing the title, keywords, and abstract. Those without obvious relevance to the research question were excluded from further analysis. Determination of exclusion occurred with conservative judgment; therefore, records of questionable status would still undergo analysis. During the evaluation of the literature, some criteria were mandatory, including mention of implicit bias and gender-based topics.

Once all publications were selected, appropriate study details were charted and stored in a database, including the primary author’s name, study design, publication year, subject (physician specialty or disease), relevant findings, and a short summary of the study. After completing the charting form, the records were classified based on connections and similarities to a variety of sub-topics within gender-based implicit bias in the clinical decision-making process. Each record received a tag to one of four sub-topics which were determined post hoc based on attributable, gendered differences in a variety of settings. (PRISMA-ScR Checklist available as a Supplementary file.)

### Group definitions

A scoping review differs from a systematic review in its intent to survey a specific field of study, identify gaps, then collate and disseminate the relevant findings; as such, there is no intention to provide a weighted, evidenced based approach in view of the results [37, 39]. After reviewing and selecting relevant literature, an analytical framework of broad, sub-topic categories was employed to further assemble the narrative presentation. The Clinical Qualities sub-topic was chosen to aggregate the studies that best represented aspects of healthcare involving medical culture, education, and other pre-clinical factors. Diagnosis of Disease and Treatment of Disease represented differences in clinical behavior and decision-making. Two discrete categories were used to describe behavioral differences in which the disease or condition was not known (Diagnosis) against when the complaint was known (Treatment). Lastly, the Outcomes sub-topic focused on the post-clinical, downstream effects of gender-based, implicit biases on modern medicine.

## Results

### Record analysis

The initial search of literature resulted in 2420 records. After screening, 162 of the records were selected for additional analysis, based on title and keyword relevance. Records were excluded from the primary round of screening, because they were the wrong subject, not focused on the delivery of healthcare, or did not study the gender of the physician. After this first round of exclusion, subsequent analysis of abstracts resulted in the rejection of 132 additional records. Of the remaining 30 records, 15 were ultimately included in the scoping review based on full-text evaluation. These 15 records were then organized into the sub-topics: 5 focused on clinical qualities, 4 on diagnosis of disease, 4 on treatment differences, and 2 on outcome differences.

### Record characteristics

The 15 records in this study were published between 2004 and 2020, with 67% majority being published after 2015 (Table 1). The origin for most of the records was the United States (60%). The remaining records either originate in Western European countries (33%) or Canada (7%). Regarding subject matter, most records focused on generalized medicine/training (53%), followed by specialty or subspecialty focused practices (47%). All records, except the systematic review by Chapman and colleagues (2013) and the perspective piece by Hemphill and colleagues (2020), were primary studies on the effects of implicit bias, gender, and the clinical decision-making process of physicians. Many of the primary research records were prospective in study design (62%); the remaining records utilized a retrospective approach through electronic health record extraction or use of publicly available and verified datasets (38%).

**Table 1:** Literature concerning implicit bias and clinical decision making

	Study Author(s)	Study Design	Study Participants	Specialty/Focus	Relevant Finding
Clinical Qualities	Amoli et al. 2016 [20]	Survey	*N* = 503	Pediatric Orthopedics	Changes in demographic make-up of pediatric orthopedics indicate higher hiring rate for females.
	Hemphill et al. 2020 [34]	Perspective	n/a	Medical Education	Physicians may acquire gender-based implicit biases through educational and formative experiences.
	Ferguson et al. 2018 [28]	Prospective validation	*N* = 247	Cardio−/Thoracic surgeons	Outcomes of clinical vignettes do not show implicit bias.
	Furnas et al. 2018 [40]	Survey	*N* = 757	Plastic surgeons	Women demonstrated a higher perception of gender concordance with their patients.
	Greene et al. 2018 [41]	Survey	*N* = 915	Clinical Preferences	Patients may have an implicit bias based solely on name when selecting a physician.
Diagnosis of Disease	Berthold et al. 2008 [42]	Cross-sectional	N = 51,053	GPs/Internists	Patients of female physicians received higher quality of care for Diabetes Mellitus Type II.
	Bouck et al. 2018 [26]	Cohort	*N* = 2394	GPs	Male physicians order more low-value tests than female physicians.
	Hamberg et al. 2004 [43]	Case Description	*N* = 289	Gastrointestinal Specialists	Physicians utilize different gender cues during the clinical work-up and diagnosis of gastrointestinal disease.
	Bernardes et al. 2013 [44]	Between Subjects	*N* = 310	GPs	Physician-held stereotypes to gender may influence the diagnosis and treatment of low-back pain.
Treatment	Daugherty et al. 2017 [32]	Prospective validation	N = 503	Cardiologists	Female physicians show lower gendered implicit bias than males.
	Hirsh et al. 2014 [35]	Analog Design (simulation)	*N* = 98	GPs	Provider sex is an influence on the selection of treatment option.
	Sabin et al. 2009 [45]	Survey	N = 2535	Medical Doctors	Only Black female physicians showed no implicit bias towards male or female patients.
	Schwartz et al. 2003 [46]	Survey	N = 289	Obesity experts	Female physicians were more likely to associate the word “fat” with bad, lazy, and stupid but not “worthless”.
Outcomes	Chapman et al. 2013 [5]	Perspective	n/a	Systematic review of literature	Implicit bias within physicians leads to perpetuating health care disparities.
	Tsugawa et al. 2017 [27]	Retrospective Analysis	*N* = 1,583,028 (episodes of care)	Internists	Female internists treat elderly hospitalized patients in a manner that lowers 30-day readmission rates and decreases hospital-related death.

*GPs, General Practitioners*

### Clinical qualities

Beginning with undergraduate and graduate medical educations, differences exist through negative perceptions, systematic favoritism, and historic biases in teaching style. Hemphill and colleagues found that female surgical residents required one-on-one facilitation while male residents performed better with repeat practice when learning surgical skills [34]. Outside of this intervention, males outperformed their female counterparts leading to a perception of increased and innate ability. In a study conducted by the American Society of Plastic Surgeons, although equally satisfied with their career choice, females were more likely to be single and not engage in hobbies when compared with their male counterparts [40]. However, measuring gender-based clinical differences is challenging. In a study of cardiothoracic surgeons, more men self-identified as “expert” and women as “competent”; yet, reviews of clinical vignettes did not show differences in their evaluations of patients [28]. Further regarding the medical work culture, an examination of how gender affects the demand and load of a surgical schedule does not reflect the equality and equity that female physicians have achieved in recent years. Female surgeons report lower salaries overall when compared with men, but this may be due in part to the decision to work less hours or take fewer cases [20]. However, one study hypothesized the salary disparity may be attributable in part to the fact that some patients make physician selections based solely on the perception of gender and race, favoring male and white sounding names despite treatment or satisfaction ratings [41].

### Diagnosis of disease

Gender-based differences exist within a variety of diagnostic performance categories. Beginning with obtaining and interpreting a health history, female physicians were more likely to factor in past medications and psychosocial health when diagnosing disease; while male physicians were more likely than female physicians to ask male patients about tobacco and alcohol use [43]. Further results found that male physicians placed more emphasis on the visibility and objectivity of patient symptoms when judging pain credibility and when making referral decisions to a psychologist or psychiatrist [44]. For certain conditions, like Diabetes Mellitus, diagnosis and prevention tend to be more comprehensive when performed by female physicians. In a large cross-sectional study (*n* = 51,053), patients of female physicians were more likely to maintain control of blood pressure, glycemic control, and blood lipoproteins [42]. Generalized diagnostic tests also show gendered differences, with older male physicians ordering more low-value screens and tests in low-risk patients (e.g., chest radiographs, Papanicolaou test, repeat dual-energy x-ray absorptiometry, and repeat electrocardiograms) compared to their female counterparts [26].

### Treatment of disease

Within the management and treatment of disease, physician gender influences a variety of factors. Female physicians were more likely to prescribe psychosocial therapies and medications than male physicians, even when bias for sexism was controlled using the Ambivalent Sexism Inventory scale [35, 47]. In the treatment of disease, evidence shows marked differences in the judgment of male physicians regarding male patients. Male patients were viewed as stronger and more open to risk regarding invasive treatments such as angiograms; however, gender of the physician was not significant when making treatment decisions after an abnormal exercise treadmill test or when recommending secondary diagnostic tests [32]. Treatment decisions may be influenced by the race of the patient, as well as the gender of the physician. In a large study of physicians (*n* = 2535) who completed the Race Attitude Implicit Associations Test [48], both genders displayed a preference for White Americans. However, when race and gender of the physician were tested, only female Black physicians displayed no implicit bias [45]. Implicit bias may translate into patient experience. All physicians surveyed reported significant anti-fat bias, but female physicians were more likely to associate the word “fat” with “bad”, “lazy”, or “stupid” [46].

### Outcomes

The effects of gender-based, implicit bias were also realized at the population level, with evidence suggesting that Medicare patients treated by male physicians have increased healthcare costs, adverse outcomes, and chance of hospital-related death compared to those treated by female physicians [27]. A study which investigated the origins of both implicit (latent) beliefs and explicit (or controlled) actions found that while more obvious acts of prejudice may evolve over time, the behavior which results from implicit bias may persist and influence decision making [5]. However, the study also found that trainings and facilitating a culture of bias literacy can aid in reducing outcome disparities in both the educational and clinical setting [5].

## Discussion

This scoping review of the literature on physician gender as a source of implicit bias suggests there are differences which exist between male and female physicians with respect to clinical decision making in some areas of practice; and these biases may manifest in behavioral performance and outcomes. When considering the role that a physician’s gender may have in the clinical decision-making process, studies which attempt to qualify causative factors were varied. Despite this challenge, this scoping review attempted to explore various manifestations within the context of medical education [34], culture [20, 40, 41], practice [26, 32, 35, 42–44], and outcomes [5, 27]. Within modern medical practice and culture, recognizing the important role of gender may serve as benefit to patient outcomes and satisfaction [49]. Despite best attempts at ensuring equity of care, accounting for physician differences, implicit biases are likely a source of continued medical disparities [28].

Reducing the burden of implicit bias is a highly researched topic with regards to the race and gender of the patient; however, this study found important gaps in the available literature regarding physician race and gender [5, 50]. Much of the literature on gender and decision-making highlights the disparities and ensuing effects on the treatment of female physicians in the workforce, and demonstrates that implicit bias may be inadvertently acquired and maintained from culture and education. One manner for reducing negative outcomes includes conceptualizing implicit bias as a manner of thinking that must first be recognized before it can be altered [51]. This can be achieved through pattern and cognitive recognition practices. Individuating, or making an active effort to focus on individual characteristics of patients, may mitigate the effects of implicit bias [5]. However, this strategy may be too time-intensive for modern medicine. Certain skills, such as self-awareness and critical reflection, can also aid in the reduction of implicit bias, thereby improving the relationship and subsequent outcomes of the patient and physician [52]. Reducing the “hidden agenda” that some medical institutions inadvertently perpetuate through structured coursework or lectures may reduce implicit biases [52]. Alternatively, trainings which include priming physicians with positive words, images, or videos could also be an adequate strategy in high-pressure or time-demanding practice situations [50].

Increasing the number of female physicians in positions of leadership may dull the pervasive impacts of implicit bias in clinical decision making by providing cognitive diversity and different viewpoints [53]. Strong role models are needed to provide a source of confidence and a pathway to leadership for younger physicians, in an environment where 44% of females see their gender as *harmful* to their status as a potential leader [54]. Through the review of literature, gender-based research is a provocative and relevant subject, evidenced by the increased number of studies published within the last decade. However, a majority of the research published in this domain focuses on patient rather than provider’s gender. This scoping review is novel in that it focuses on the physician’s gender as a contributing factor to clinical practice.

In interpreting the results of this scoping review, however, some limitations should be considered. First, despite attempts to perform an extensive and comprehensive search, there may be records that were never identified or inadvertently removed from the review process. Additionally, no unpublished studies were included in this review, so it is possible that new research on implicit bias could exist. Secondly, filtering for the reviewed articles required several necessary terms be present, including implicit bias and gender-based phrasing. This may have been too limiting, as there are alternative ways of describing implicit bias without overtly mentioning it. Lastly, as in many domains where more positive or provocative results are desired, there is a risk for publication bias. Our search terms could have influenced the publications ultimately selected for this scoping review, or there may be a lack of publications showing opposite effects.

## Conclusion

Despite these shortcomings, the findings from this scoping review suggest that gender-based implicit bias may affect the decision-making processes of physicians in the real world. These findings pertain to the diagnosis, treatment, and overall outcomes of disease. However, much is still unknown about the effect of the physician’s gender on their clinical decision-making processes. Further studies could explore outcome differences between recent graduates and career physicians, for both female and male physicians. Future research should explore differences in decision making between male and female physicians in healthcare environments where female role models are present in positions of leadership in order to test hypothesized changes in behavior.

## Supplementary Information

**Additional file 1.**

**Additional file 2.**

## Data Availability

Datasets generated during this study are included in the published article.

## Abbreviations

AAMC: Association of American Medical Colleges
GP: General practitioner
MeSH: Medical subject heading
