# Supplementary material for: Physician gender as a source of implicit bias affecting clinical decision-making processes: a scoping review
Source: BMC Med Educ. 2021 Mar 19;21:171. doi: 10.1186/s12909-021-02601-2 (PMC7980423; doi:10.1186/s12909-021-02601-2)
Supplement: Supplementary file 2 — Additional file 2. [file 12909_2021_2601_MOESM2_ESM.docx]

**Appendix A. Complete list of Search Keywords**

| "physician gender" OR  "physician sex" OR  "male physician" OR  "male physicians" OR  "men physicians" OR  "Physicians, Women"[Mesh] OR  "female physician" OR  "female physicians" OR  "women physicians" OR  "men physicians" OR  "Practice Patterns, Physicians'"[Mesh] OR  "physician practice patterns" OR  "physician's practice patterns” OR  "clinical practice patterns" OR  "health care professional" OR  "health care professionals" OR  "doctor" OR  "doctors" | AND  "gender concordance" OR  "sex difference" OR  "gender difference” OR  "sex differences" OR  "gender differences" OR  "Sex Characteristics"[Mesh] OR  "sex characteristic" OR  "sex characteristics" OR  "sex-based difference" OR  "sex-based differences" OR  "gender effect" OR  "gender effects" | AND  "Decision Making"[Mesh] OR  "decision making" OR  "decision makings" OR  "Clinical Decision-Making"[Mesh] OR  "Physician-Patient Relations"[Mesh] OR  "Sex Factors"[Mesh] OR  "sex factors" OR  "sex factor" | AND  "Bias"[Mesh] OR  "bias" OR  "biased" OR  "prejudice" OR  "prejudiced" |
| --- | --- | --- | --- |
